# A Potent Inhibitor of SIK2, 3, 3′, 7-Trihydroxy-4′-Methoxyflavon (4′-*O*-Methylfisetin), Promotes Melanogenesis in B16F10 Melanoma Cells

**DOI:** 10.1371/journal.pone.0026148

**Published:** 2011-10-13

**Authors:** Ayako Kumagai, Nanao Horike, Yudai Satoh, Tatsuya Uebi, Tsutomu Sasaki, Yumi Itoh, Yoshiyuki Hirata, Kozue Uchio-Yamada, Kazuo Kitagawa, Shinichi Uesato, Hidehisa Kawahara, Hiroshi Takemori, Yasuo Nagaoka

**Affiliations:** 1 Department of Life Science and Biotechnology, Faculty of Chemistry, Materials and Bioengineering, Kansai University, Osaka, Japan; 2 Laboratory of Cell Signaling and Metabolic Disease, National Institute of Biomedical Innovation, Osaka, Japan; 3 Department of Neurology, Osaka University Graduate School of Medicine, Osaka, Japan; 4 Laboratory of Animal Models for Human Diseases, National Institute of Biomedical Innovation, Osaka, Japan; Roswell Park Cancer Institute, United States of America

## Abstract

Flavonoids, which are plant polyphenols, are now widely used in supplements and cosmetics. Here, we report that 4′-methylflavonoids are potent inducers of melanogenesis in B16F10 melanoma cells and in mice. We recently identified salt inducible kinase 2 (SIK2) as an inhibitor of melanogenesis via the suppression of the cAMP-response element binding protein (CREB)-specific coactivator 1 (TORC1). Using an *in vitro* kinase assay targeting SIK2, we identified fisetin as a candidate inhibitor, possibly being capable of promoting melanogenesis. However, fisetin neither inhibited the CREB-inhibitory activity of SIK2 nor promoted melanogenesis in B16F10 melanoma cells. Conversely, mono-methyl-flavonoids, such as diosmetin (4′*-O-*metlylluteolin), efficiently inhibited SIK2 and promoted melanogenesis in this cell line. The cAMP-CREB system is impaired in *A^y^/a* mice and these mice have yellow hair as a result of pheomelanogenesis, while *Sik2^+/−^*; *A^y^/a* mice also have yellow hair, but activate eumelanogenesis when they are exposed to CREB stimulators. Feeding *Sik2^+/−^*; *A^y^/a* mice with diets supplemented with fisetin resulted in their hair color changing to brown, and metabolite analysis suggested the presence of mono-methylfisetin in their feces. Thus, we decided to synthesize 4′*-O-*methylfisetin (4′MF) and found that 4′MF strongly induced melanogenesis in B16F10 melanoma cells, which was accompanied by the nuclear translocation of TORC1, and the 4′*-O-*methylfisetin-induced melanogenic programs were inhibited by the overexpression of dominant negative TORC1. In conclusion, compounds that modulate SIK2 cascades are helpful to regulate melanogenesis via TORC1 without affecting cAMP levels, and the combined analysis of *Sik2^+/−^* mice and metabolites from these mice is an effective strategy to identify beneficial compounds to regulate CREB activity *in vivo*.

## Introduction

Melanin plays an important role in animals by preventing the cellular damage induced by ultraviolet (UV) light. When keratinocytes in the skin are exposed to UV irradiation, alpha-melanocyte stimulating hormone (alpha-MSH), a peptide hormone, is processed from the precursor peptide proopiomelanocortin and is secreted as a paracrine factor [1], [2], [3], [4]. Secreted alpha-MSH subsequently binds to its receptor, the melanocortin 1 receptor, on the membrane of melanocytes and activates adenylyl cyclase, resulting in increased levels of intracellular cAMP. cAMP then activates protein kinase A (PKA), which phosphorylates the transcription factor cAMP response element (CRE)-binding protein (CREB) at Ser133, initiating the transcriptional cascades of the melanogenic program, *e.g*., the induction of microphthalmia-associated transcription factor (*Mitf*) expression [5], [6]. Finally, MITF induces the expression of tyrosinase, which initiates the catalysis of melanin from tyrosine by the sequential hydroxylation [7].

Flavonoids are polyphenolic compounds that are widely distributed in vegetables and fruits and protect organisms from damage caused by UV exposure and reactive oxygen species [8], [9]. Flavonoids consist of two parts: one is a basic skeleton having three rings (A, B, and C) with one or two oxygen molecules (*e.g.*, flavan or flavone, respectively), while the other part consists of modified side chains, *e.g*., hydroxy, methoxy, and *O-*glycosyl groups [10].

Based on the health-promoting effectiveness of flavonoids and their low levels of toxicity, they are used as supplements to prevent disease, such as cancer and metabolic syndromes. In addition, flavonoids, *e.g.*, procyanidins [11] and quercetin [12], are added to cosmetic products to suppress melanogenesis by inhibiting tyrosinase. However, other flavonoids have been reported to have the opposite effect on melanogenesis. For example, nobiletin [13] was shown to stimulate melanogenesis by upregulating the extracellular signal-regulated kinase (ERK) pathway, which induces the expression of tyrosinase via the activation of CREB. In addition to this pathway, nobiletin inhibits phosphodiesterase leading to an elevation of intracellular cAMP levels [14], which bypasses the alpha-MSH pathways.

We previously found that the CREB-specific coactivator TORC1 (transducer of CREB activity, also called CRTC1) and its repressor, salt-inducible-kinase 2 (SIK2) [15], [16], [17], are fundamental determinants of the melanogenic program in mice [18]. Exposure of B16F10 melanoma cells to UV light results in the immediate nuclear translocation of TORC1, which is inhibited by SIK2. Overexpression of dominant negative TORC1 also inhibits UV-induced *Mitf* gene expression and melanogenesis. alpha-MSH signaling regulates hair pigmentation, and a decrease in alpha-MSH activity in hair follicle melanocytes switches the synthesis of melanin from eumelanin (black) to pheomelanin (yellow). Mice with the lethal yellow allele of agouti (*A^y^/a*) have yellow hair due to the impaired activation of the alpha-MSH receptor. *A^y^/a* mice with *Sik2* ^−/−^ have brown hair, indicating that SIK2 represses eumelanogenesis in mice.

Here we report that flavonoids with an *O-*methyl group at their 4′ position efficiently inhibit SIK2 action in cultured melanoma cells and promote the melanogenic program in a TORC1-dependent manner. Diosmetin (4′-*O*-methylluteolin) and fisetin (after its conversion into 4′-*O*-methylfisetin *in vivo*) enhance eumelanogenesis in *A^y^/a* mice whose CREB-cascades were sensitized by the *Sik2* heterozygous (*Sik2* ^+/−^) background.

## Results

### SIK2 inhibitory activity of the flavonoids

To identify SIK2 inhibitory substances, we employed an enzyme-linked immunosorbent assay (ELISA) system and screened the compounds using a kinase inhibitor library (BioMol). Most candidates, *e.g*., staurosporine, hypericin, etc. [19], were nonspecific kinase inhibitors and were considered difficult to utilize in structure-activity-related studies. However, quercetin, a flavonoid, has a number of derivatives despite its weak inhibitory activity (IC_50_ = 500 nM); therefore, we decided to examine the SIK2-inhibitory activity of quercetin derivatives.

**Figure 1:**
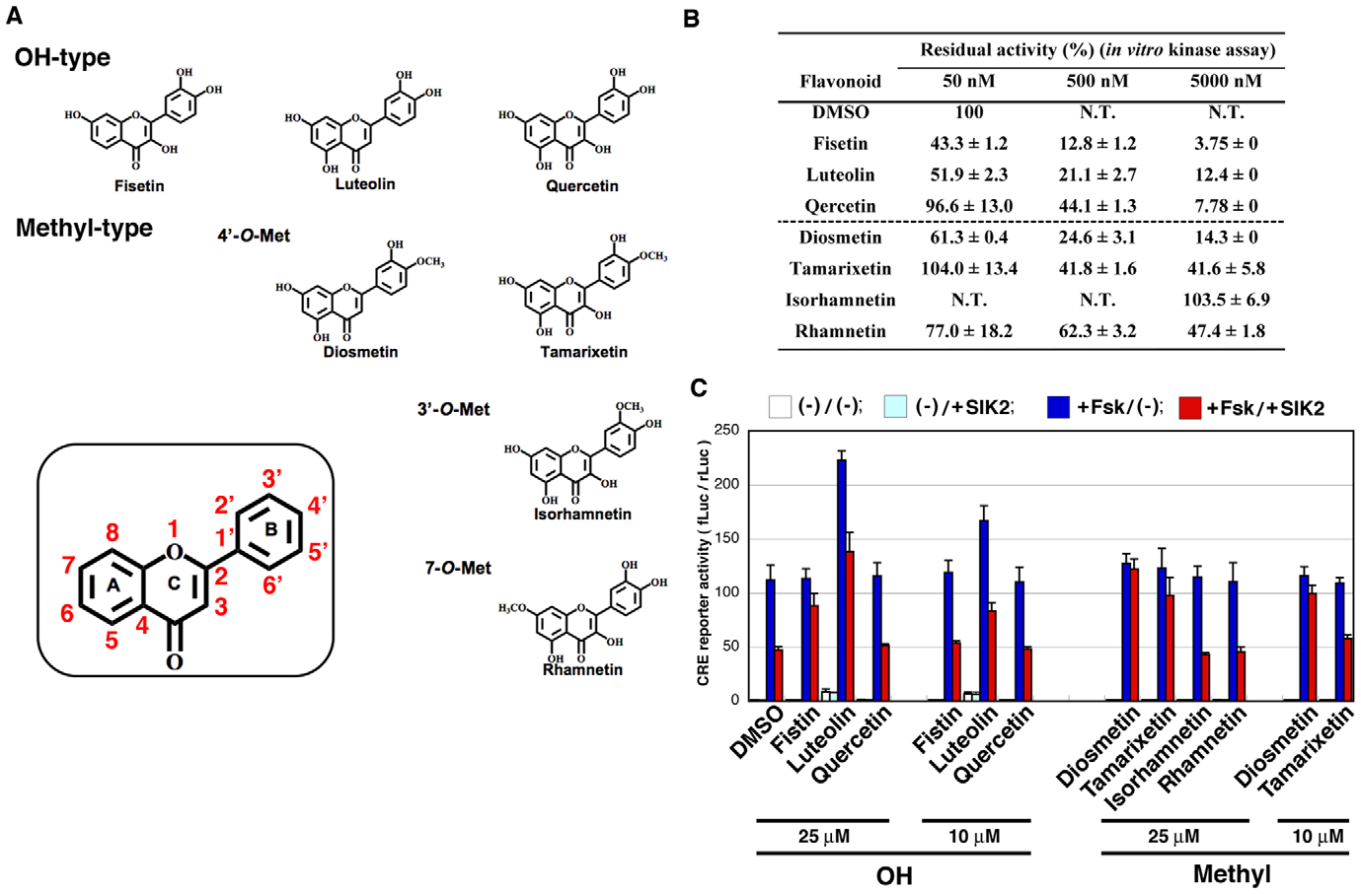
Inhibition of SIK2 by flavonoids. (A) Structure of the flavonoids used in this study. (B) *In vitro* kinase assay of SIK2. GST-SIK2 expressed in COS-7 cells was used as the enzyme, while GST-TORC2 peptide [42], expressed in *Escherichia coli*, was used as the substrate for the ELISA. The optical density (OD) value in the absence of a flavonoid was set as 100%. n = 2, means and differences are shown. (C) HEK293 cells transformed with the CRE-Luc firefly luciferase plasmid (200 ng) and pRL-Tk Int- (internal *Renilla* luciferase: 30 ng) in the presence or absence of pTarget-SIK2 (50 ng) were treated with forskolin (Fsk: 20 µM) in the presence of the indicated dose of flavonoids. The ratio of firefly luciferase to *Renilla* luciferase is shown. n = 2, means and differences are shown.

The structures of flavonoids (Figure 1A) and their SIK2-inhibitory activity (Figure 1B) are shown. Fisetin was found to inhibit SIK2 even at a low concentration (50 nM). Some of the *O*-methylated derivatives, such as diosmetin, inhibited SIK2 at medium concentrations (50–500 nM).

To monitor the SIK2-inhibitory activity in cultured cells (HEK293), we employed the CRE-reporter assay. As shown in Figure 1C, 25 µM fisetin inhibited the SIK2-mediated suppression of CRE activity that had been upregulated by the cAMP-agonist forskolin. However, a low dose of fisetin (10 µM) failed to inhibit SIK2 activity in cultured cells, while diosmetin was able to inhibit SIK2 even at a low dose (10 µM), suggesting that other parameters, such as cell permeability, may affect their SIK2-inhibitory activity in cultured cells. On the other hand, it is also important that the *O*-methyl group at the 4′-position of the B-ring more increased their SIK2-inhibitory activity in cultured cells than the *O*-methyl group at the 3′-position or at the 7- position.

### *O*-methyl-flavonoids promote melanogenesis in B16F10 cells

**Figure 2:**
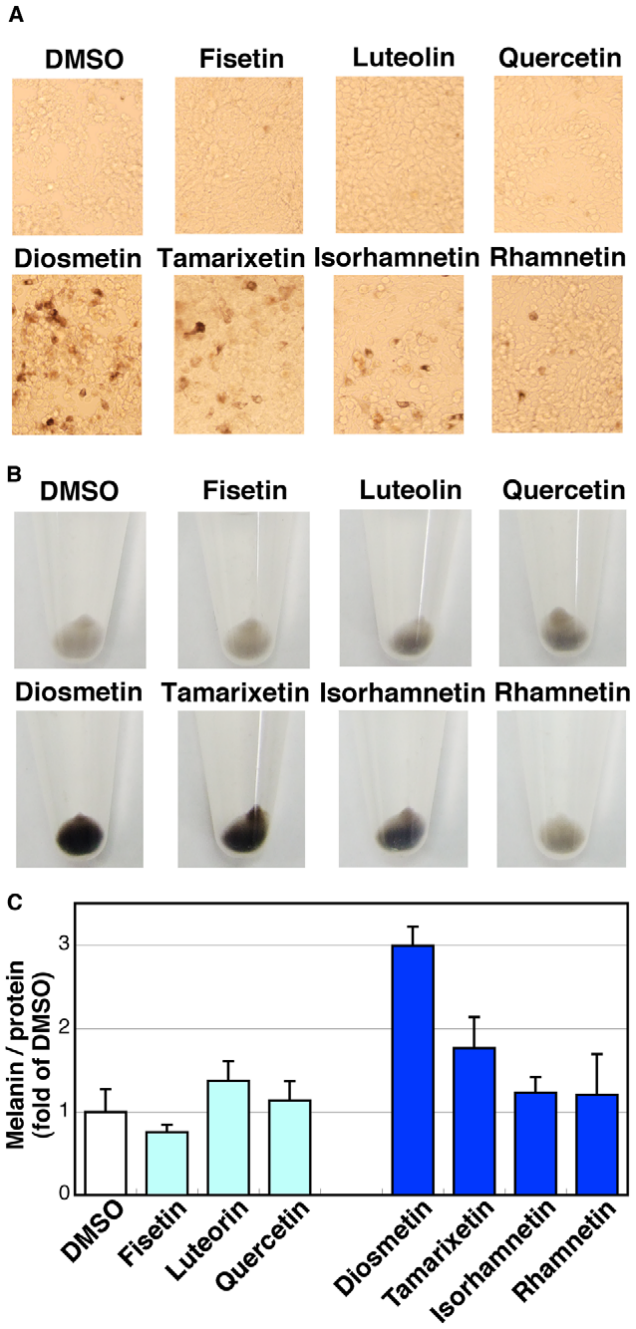
Induction of melanogenesis by flavonoids in B16F10 melanoma cells. (A) B16F10 melanoma cells were treated with 10 µM flavonoids for 3 d with a medium change at day 2. (B) The cells were recovered in test tubes. (C) Melanin was extracted with an alkaline method. After normalization of the melanin content to the protein amount in each sample, the melanin level was expressed as fold of control (DMSO-treated cells). n = 3, means and standard deviations (S.D.) are shown.

Because one of the representative phenomena of SIK2 inhibition is the promotion of melanogenesis, we employed a melanogenesis assay using B16F10 melanoma cells to evaluate SIK2-inhibitory flavonoids. As shown in Figure 2, non-methylated flavonoids did not induce melanogenesis. In contrast, the 4′-*O*-methyl flavonoids diosmetin and tamarixetin efficiently induced melanogenesis, while the 3′-*O*-methyl flavonoid isorhamnetin had a modest effect. A small induction of melanogenesis was observed when the 7-*O*-methyl flavonoid rhamnetin was added into the cultured medium.

The requirement of the methyl group at the 4′-position of the B-ring for melanogenesis in B16F10 melanoma cells was similar to that for the inhibition of the SIK2-mediated suppression of CREB activity in HEK293 cells, suggesting that 4′-*O*-methyl flavonoids may induce melanogenesis mainly due to the inhibition of SIK2. The effect of fisetin on melanogenesis was not affected by other factors, such as cell-permeability and stability, which are different between cell types, because fisetin did not affect CREB activity in B16F10 melanoma cells (shown later).

### Flavonoids promote eumelanogenesis *in vivo*

CREB activity determines the ratio of eumelanogenesis to pheomelanogenesis in hair follicle melanocytes *in vivo*, and inhibition of SIK2 facilitates eumelanogenesis due to the constitutive activation of CREB. Mice with the *lethal yellow* allele of *agouti* (*A^y^*) have yellow hair due to the impaired activation of the alpha-MSH receptor followed by the inactivation of the cAMP-CREB cascade. The *Sik2^−/−^* genetic background reactivates the CREB cascade in *A^y^/a* mice, which restores the yellow hair color to wild-type mice (brown).

**Figure 3:**
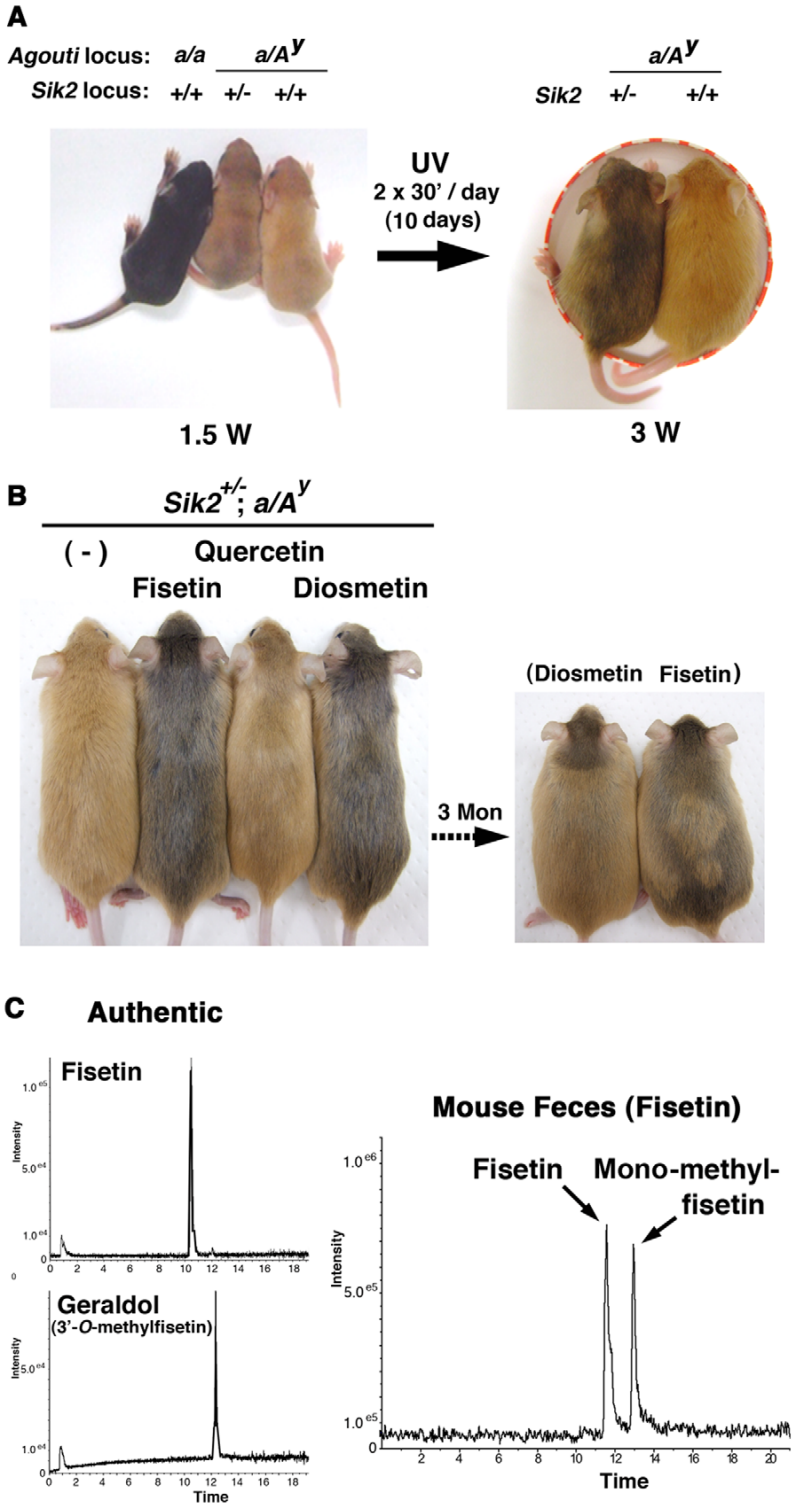
Induction of melanogenesis by flavonoids *in vivo*. (A) 1.5-week-old *A^y^/a* male mice with different *Sik2* backgrounds (*Sik2* ^+/+^ or *Sik2* ^+/−^) exposed to a black lamp (15 W at 20 cm distance) for 30 min twice daily for 10 d. (B) 4-week-old male mice were fed with a diet supplemented with 0.2% flavonoids. After 1 week, the diet was changed to a normal diet (flavonoid free), and the mice were fed for an additional week until all of their hair was replaced by newly grown hair. After a photograph was taken under anesthesia, the mice were fed for a further 3 months until the next set of hair grew. The photographs show a representative mouse from each group (n = 4). (C) Flavonoids in feces derived from fisetin-treated mice were extracted with ethyl acetate and detected by LC-MS with a scan range of ms 285–299, as described in the Materials and Methods. The positions of authentic flavonoids are also shown in left panels.

The *Sik2* heterozygous (*Sik2^+/−^*) background partially restored hair color, but *Sik2^+/−^*; *A^y^/a* mice were highly sensitive to CREB agonists, such as UV irradiation, which appeared as a hair color change (Figure 3A). Therefore, we decided to use *Sik2^+/−^*; *A^y^/a* mice to evaluate the effect of flavonoids on melanogenesis *in vivo*. We assessed the activity of fisetin, quercetin, and diosmetin because their low cost would facilitate their use as dietary supplements. As shown in Figure 3B, fisetin and diosmetin changed the hair color of *Sik2^+/−^*; *A^y^/a* mice, while quercetin had a modest effect. This hair color change was reversible. The difference between fisetin and quercetin could be explained by their inhibitory efficiency toward SIK2 in HEK293 cells (Figure 1C); however, the fact that fisetin promoted eumelanogenesis at the same level as diosmetin disagreed with the results observed in B6F10 melanoma cells (Figure 2). Therefore, we surmised that some of the metabolites, probably *O-*methylfisetin, might promote eumelanogenesis in mice consuming fisetin. To confirm this hypothesis, we analyzed fisetin metabolites in feces and identified mono-methylfisetin in the metabolites (Figure 3C).

### 4′-*O*-methylfisetin strongly promotes melanogenesis in B16F10 melanoma cells

**Figure 4:**
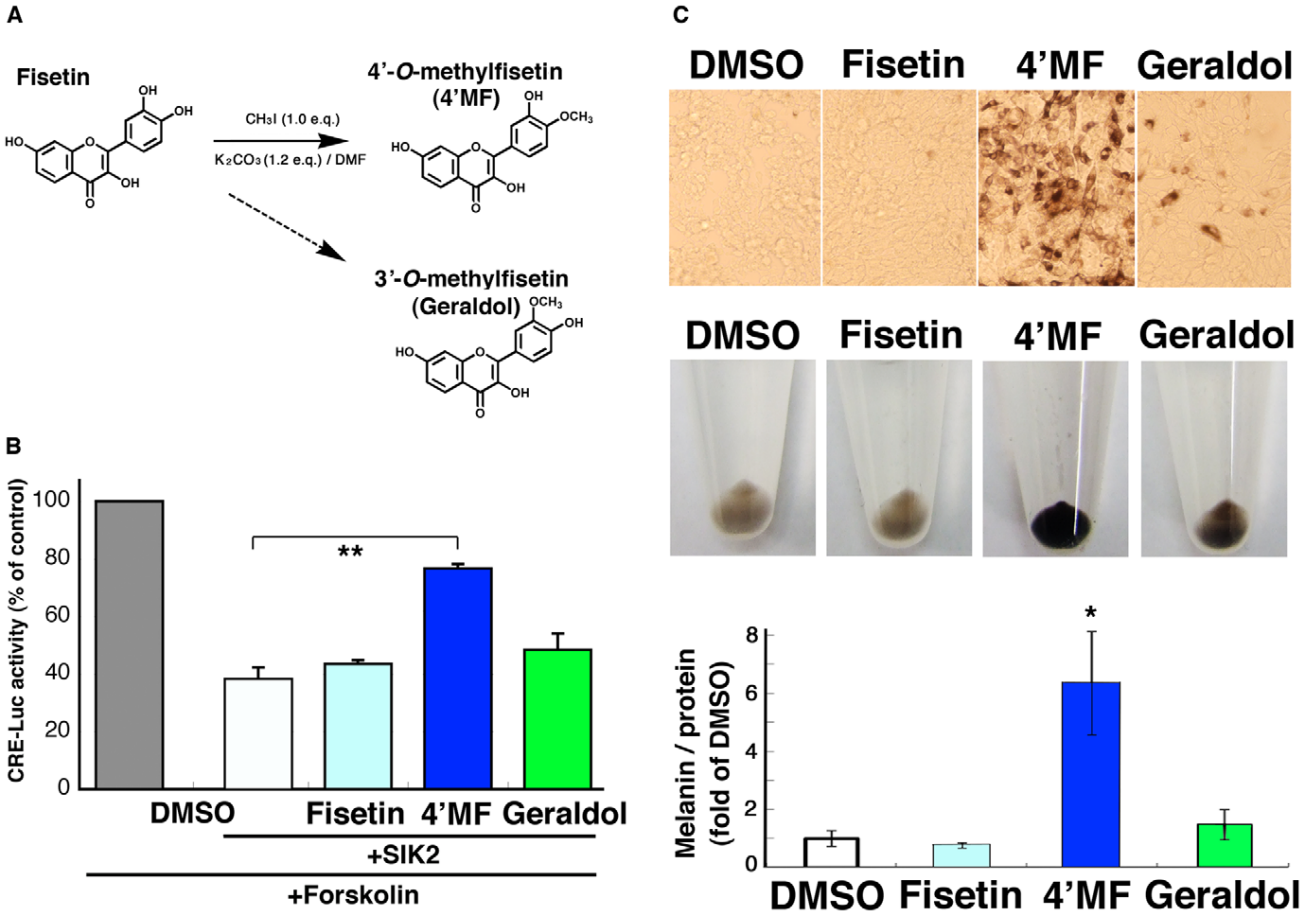
4′-*O-*methylfisetin (4′MF) inhibits SIK2-mediated CRE suppression and induces melanogenesis in B16F10 melanoma cells. (A) Synthesis of 4′MF. (B) B16F10 melanoma cells transformed with CRE-Luc firefly luciferase plasmid (200 ng) with pRL-Tk Int- (internal *Renilla* luciferase: 30 ng) in the presence or absence of pTarget-SIK2 (50 ng) were treated with forskolin (Fsk: 20 µM) in the presence of the indicated dose of flavonoids. The relative units of firefly luciferase were normalized to *Renilla* luciferase, and expressed as % of control (without flavonoid or SIK2). n = 3, means and S.D. are shown. **, *p<0.01*. (C) B16F10 melanoma cells were treated with 10 µM flavonoids for 3 d, with a medium change at day 2, and melanin was measured. n = 3, means and S.D. are shown. *, *p<0.05*.

The LC system is not able to separate 4′-*O*-methyl flavonoids from their 3′-*O*-methyl isomers, and 4′-*O*-methylfisetin is not commercially available, while 3′-*O*-methyl fisetin is available as geraldol. Therefore, we decided to synthesize 4′-*O*-methylfisetin using CH_3_I to confirm its potential as a promoter of melanogenesis (Figure 4A). The 4′-OH group of fisetin might more actively accept the methyl group than the 3′-OH group did because the yield of 4′-*O*-methylfisetin (5.3%, 5.9 mg) was higher than the 3′-*O*-isomer (<1.0%). The identity of 4′-*O*-methylfisetin was confirmed by ^1^H NMR, ^13^C NMR, and ESI-MS [20] (Figure S1).

When 4′-*O*-methylfisetin was added into the culture medium of B16F10 melanoma cells, the SIK2-mediated suppression of CREB activity was weakened (Figure 4B) and melanogenesis was strongly promoted (Figure 4C), suggesting that eumelanogenesis in fisetin-treated mice might be induced by 4′-*O*-methylfisetin.

### 4′-*O*-methylfisetin promotes melanogenesis dependent on TORC1 and independent of cAMP

**Figure 5:**
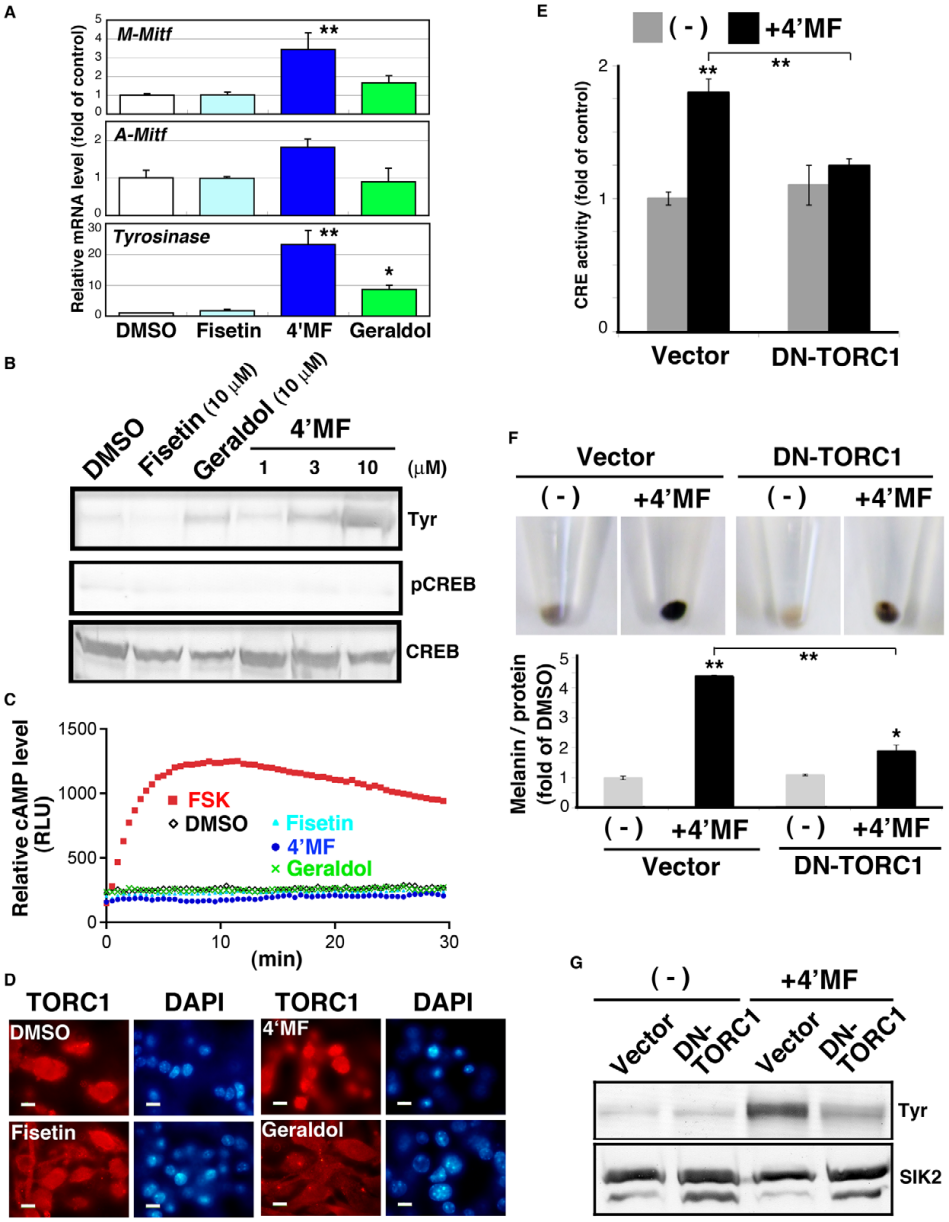
4′MF induces melanogenesis by activating TORC1 without enhancing the cAMP level in B16F10 melanoma cells. (A) Quantitative PCR analyses were performed with total RNA prepared from flavonoid-treated B16F10 melanoma cells (10 µM for 3 d, with a medium change at day 2). The mRNA levels are shown as fold of control. n = 3, means and S.D. are shown. 4′MF: 4′-*O*-methylfisetin. * and **, *p<0.05 and <0.01*, respectively. (B) Tyrosinase (Tyr) protein was detected by western blot analysis. 4′MF was added at the indicated concentration. pCREB (pSer133) and total CREB were also examined using the same cell lysate. The panels represent the findings from one of the duplicated experiments. (C) B16F10 cells transformed with the cAMP-indicator plasmid pGloSensor-22F were treated with 10 µM flavonoid or forskolin (Fsk: 20 µM) in the presence of luciferin. The relative light units are shown as relative cAMP levels. (D) B16F10 cells were treated with 10 µM flavonoids for 72 h and then fixed with 4% paraformaldehyde. TORC1 was detected with the anti-TORC1/3 antibody. Nuclei were stained with DAPI. (E) B16F10 melanoma cells transfected with the dominant negative TORC1 (DN-TORC1) adenovirus or empty adenovirus (Vector) were transformed with the CRE-Luc firefly luciferase plasmid (200 ng) and pRL-Tk Int- (internal *Renilla* luciferase: 30 ng). After 24 h, the cells were treated with 10 µM 4′MF for an additional 24 h. CRE activity was expressed as fold of control (the cells were transfected with the empty adenovirus and not treated with 4′MF). n = 3, means and S.D. are shown. Bars indicate 10 µm. (F) B16F10 melanoma cells transfected with the adenoviruses as in (E) were treated with 10 µM 4′MF for 3 d with a medium change at day 2, and the melanin content was measured. n = 3, means and S.D. are shown. (G) Tyrosinase protein levels in B16F10 melanoma cells (the same sample as in F) were examined by western blot analyses. SIK2 was detected as a loading control.

To examine the molecular mechanisms underlying 4′-*O*-methylfisetin-induced melanogenesis, we monitored the mRNA expression of the melanogenic genes, M-type *Mitf*, A-type *Mitf*, and *Tyrosinase*. As shown in Figure 5A, 4′-*O*-methylfisetin induced these mRNAs in B16F10 melanoma cells. The Tyrosinase protein level (Tyr) was also elevated in 4′-*O*-methylfisetin-treated cells (Figure 5B), which was observed from 3 µM. Geraldol was also able to induce Tyrosinase expression, but its efficiency was less than one-third of 4′-*O*-methylfisetin.

When we examined CREB phosphorylation levels (Figure 5B), we noticed that 4′-*O*-methylfisetin induced melanogenesis without elevating the phosphorylation of CREB at Ser133. This was confirmed by an assay indicating that these flavonoids had little effect on intracellular cAMP levels in B16F10 melanoma cells (Figure 5C). In addition to cAMP/PKA cascade, 4′-*O*-methylfisetin did not alter the phosphorylation levels of Erk and GSK-3beta, while fisetin and geraldol enhanced pGSK-3 beta signals (Figure S2).

Since the loss of SIK2 induces melanogenesis by activating TORC1, we monitored the activation of TORC1 by its intracellular distribution. As shown in Figure 5D, 4′-*O*-methylfisetin induced the nuclear accumulation of TORC1, but other flavonoids did not. We then examined whether 4′-*O*-methylfisetin was able to activate CREB, and if so, whether this activation was dependent on TORC1. Expectedly, 4′-*O*-methylfisetin upregulated CRE-reporter activity, which was inhibited by the overexpression of DN-TORC1.

Finally, we tested whether DN-TORC1 was able to inhibit 4′-*O*-methylfisetin-induced melanogenesis. As shown in Figure 5E, DN-TORC1 inhibited melanin synthesis (Figure 5F), which was accompanied by the suppression of Tyrosinase expression (Figure 5G). These results suggest that 4′-*O*-flavonoids, especially 4′-*O*-methylfisetin, are potent inhibitors of SIK2 and capable of activating TORC1 followed by the induction of the melanogenic program in mice.

## Discussion

We have shown that 4′*-O-*methyl flavones can inhibit SIK2 activity and promote melanogenesis via the activation of TORC1 in B16F10 melanoma cells [18]. However, first, we have to discuss about the discrepancy found between the *in vitro* and cultured cell assays for structure activity correlation. The *in vitro* kinase assay using the TORC peptide suggested that non-methylated flavones more potently inhibited SIK2 than their methylated derivatives. However, in HEK293 cells and B16F10 melanoma cells, 4′*-O-*methylflavone inhibited SIK2 more efficiently, suggesting several mechanisms exist by which flavones can inhibit SIK2 in cultured cells. This hypothesis is also supported by the observation that 3′*-O-*methylflavones, such as isorhamnetin and geraldol, do not inhibit SIK2 *in vitro*, while they weakly induce melanogenesis in B16F10 melanoma cells. These results suggested that methylated flavonoids induce the melanogenic program by several mechanisms, such as enhanced cell permeability and SIK2-independent signaling pathways.

Meanwhile, it was also true that the efficiency of SIK2 inhibition and the potency of melanogenic promotion by 4′*-O-*methylflavones in cultured cells (4′-*O*-methylfisetin *>* diosmetin >tamarixetin) correlated well with the efficiency of SIK2-kinase inhibition *in vitro* by their non-methylated cognates (fisetin > luteolin > quercetin). Moreover, fisetin promoted eumelanogenesis in *A^y^/a*; *Sik2^+/−^* mice more potently than quercetin, suggesting that a synergistic effect between the direct inhibition of SIK2 by a structural dependence of flavones and an indirect effect via a mechanism depending on their 4′*-O-*methoxy groups may efficiently promote melanogenesis in mice.

A number of factors and related compounds have been reported to intricately modulate the melanogenic program. For example, tyrosine kinases and glycogen synthase kinase 3 beta (GSK-3 beta) play opposing roles in the regulation of melanogenesis in melanocytes [21]. The transcriptional activity of the MITF protein is modulated by protein kinase cascades that are induced by the stem cell factor and its receptor kinase c-KIT. The activation of c-KIT invokes two opposing pathways: the RAS-RAF-MEK and PI3K-AKT pathways. The RAS-RAF-MEK pathway activates ERK-p90RSK, which phosphorylates CREB at Ser133 and MITF at Ser73 and Ser409 [22] and promotes melanogenesis, whereas AKT inhibits the MITF-activating kinase GSK-3 beta and downregulates melanogenesis [23]. The plant steroid diosgenin also inhibits melanogenesis by activating PI3K signaling [24].

However, the action of GSK-3 beta in the regulation of melanogenesis is complicated and paradoxical. The promoter activity of the *Mitf* gene is upregulated by the beta-catenin-TCF/LEF complex [25], and the phosphorylation of beta-catenin by GSK-3 beta [26] destabilizes beta-catenin and leads to the suppression of MITF-induced melanogenesis [27]. The observation that indirubin derivatives, potent inhibitors of GSK-3 beta [27], [28], stabilize the beta-catenin-TCF/LEF complex and promote melanogenesis in B16F10 melanoma cells suggests that *Mitf* expression, rather than the phosphorylation-dependent activation of MITF, is the rate-limiting step of the melanogenic program [29].

The GSK-3 beta-mediated regulation of melanogenesis is often accompanied by the activation of the cAMP-PKA-CREB pathway. The plant steroid glycyrrhizin inhibits GSK-3 beta activity, while stimulating CREB-mediated transcription by activating PKA, which results in the promotion of melanogenesis [30]. Meanwhile, we reported that the GSK-3 beta inhibitor indirubin induces the degradation of SIK1 and SIK2 proteins in COS-7 cells [31] and in differentiating C2C12 myocytes [32]. GSK-3 beta is capable of phosphorylating (activating) sites in the activation loop of SIK1/2, and the activated SIK1/2 proteins are stable [31], suggesting that B16F10 melanoma cells that have been treated with GSK-3 beta inhibitors have low levels of SIK2, which would promote melanogenesis. Meanwhile, 4′-*O*-methylfisetin did not modulate the AKT-GSK-3 beta and MEK cascades, suggesting that the melanogenic programs induced by 4′*-O-*methylflavones may be different from those induced by plants compounds modulating the AKT-GSK-3 beta and MEK cascades.

Some methylated flavonoids, such as nobiletin [14] and ayanin [33], inhibit phosphodiesterase, which increases the intracellular cAMP levels [34]. In contrast to these polymethylated flavonoids, 4′-*O*-methylfisetin elevates neither CREB phosphorylation levels nor cAMP-indicator luciferase activity, irrespective of the length of treatment, suggesting that 4′-*O*-methylfisetin upregulates CREB activity independently of cAMP. The mechanism of 4′-*O*-methylfisetin-induced CREB activity may depend on the activity of TORC1 induced by SIK2 inhibition.

TORC1, or its other isoforms, plays important roles in neuronal activity, such as memory in the hippocampus [35], [36], behavior (food intake) in the arcuate and ventromedial nuclei [37], and corticotrophin-releasing hormone synthesis in the hypothalamus [38]. In addition to these roles, we also found that TORC1 is essential for neuronal survival after brain ischemia [19], which is evident in *Sik2^−/−^* mice. Interestingly, fisetin was found to enhance memory function in the brain and long term potentiation in cultured PC12 cells via MEK-ERK-mediated CREB activation [39]. Because 4′-*O*-methylfisetin did not activate ERK in B16F10 melanoma cells, the upregulation of TORC activity by SIK2 inhibition has been suggested be a beneficial strategy for the treatment of neuronal diseases, and fisetin or 4′-*O*-methylfisetin may be helpful to perform this strategy.

On the other hand, the present study also revealed that heterozygous insufficiency of the *Sik2* allele increases the sensitivity of CREB-mediated gene expression *in vivo*, such as switching to eumelanogenesis in hair melanocytes. This phenomenon may be helpful to screen CREB activators *in vivo*. Given that the daily food intake of *A^y^/a* mice is ∼4 g on average, the present dose of fisetin, 400 mg/kg, is not extremely high. Unfortunately, fisetin intake elevates the blood glucose levels of *A^y^/a* mice, while diosmetin did slightly (data not shown). As there was no significant difference in blood glucose levels between wild-type and *Sik2^−/−^* mice [18], [40], fisetin may affect blood glucose homeostasis in a SIK2-independent manner.

In conclusion, by modulating SIK2 signaling, we were able to identify a biologically active substance, 4′-*O*-methylfisetin, which initiated CREB-mediated transcription via TORC1 activation. In this study, we also found that the hair color of *Sik2^+/−^* mice and the analysis of metabolites in their feces and blood may act as beneficial indicators to develop compounds that modulate CREB activity.

## Materials and Methods

### Flavonoids

Luteolin, diosmetin, quercetin, tamarixetin, isorhamnetin, rhamnetin, and geraldol were obtained from Extrasynthese (Genay Cedex, France). Fisetin and forskolin were purchased from Wako Pure Chemicals Co. Ltd., (Osaka, Japan) and Sigma-Aldrich (St. Louis, MO, USA), respectively. These compounds were dissolved in dimethyl sulfoxide (DMSO) as ×1000 stock solutions.

### Cell culture, flavonoid treatment, and melanin measurement

B16F10 murine melanoma cells and HEK293 cells were obtained from the American Type Culture Collection (Manassas, VA, USA). B16F10 cells were growth at 37°C under 5% CO_2_ in Dulbecco's modified Eagle's medium (DMEM; high glucose) (Wako) supplemented with 10% fetal bovine serum (FBS), penicillin (100 U/mL), and streptomycin (50 µg/mL). HEK293 cells were growth at 37°C under 5% CO_2_ in DMEM (low glucose) (Wako) supplemented with 10% FBS and penicillin/streptomycin.

B16F10 were seeded in 6-well plates at a density of 3.4×10^5^ cells/well. After 24 h, the culture medium was replaced with fresh medium supplemented with flavonoids, and, after 48 h, the medium was changed again with fresh medium containing the same flavonoids. After an additional 24 h, the cells were harvested for the melanin or mRNA/protein assays.

To measure melanin, the cells were washed twice with phosphate-buffered saline (PBS), suspended in PBS, and recovered by centrifugation at 8,000 rpm for 1.5 min. The cell pellet was suspended in 300 µL of 1 N NaOH and incubated at 45°C for 2 h, and, then, melanin was extracted with a chloroform-methanol mixture (2∶1). Melanin was detected with a spectrophotometer (BIO-RAD Model 680 MICRO PLATE READER; Bio-Rad, Hercules, CA, USA) at 405 nm. The standard curve was obtained by using purified melanin (0–1000 µg/mL). The protein concentration of the cell pellets was determined using the Bradford reagent (Bio-Rad) and used for normalization of the melanin content.

### Animal experiments and liquid chromatography-mass spectrometry (LC-MS) analysis of flavonoids

The experimental protocols for mice were approved by the committee at the National Institute of Biomedical Innovation (approval ID: DS20-55). *Sik2^+/−^*; *A^y^* mice (4-week-old male mice; the mice were gifts from ProteinExpress Co. Ltd., Chiba, Japan) were housed under standard light (08:00–20:00) and temperature (23°C/60% humidity) conditions.

Mice feces (3.0 g dry weight) were soaked in water : ethyl acetate (1∶1), and the flavonoids were recovered from the organic phase. Ethyl acetate was evaporated under N_2_ gas, and the dried residues were dissolved in 25% acetonitrile/water and subjected to LC-MS analysis (API 3000 mass spectrometer; Applied Biosystems, Foster City, CA, USA). To separate the flavonoids, a C_18_ column (2.0×50 mm i.d., particle size 5 µm) (Nacalai tesque, Kyoto, Japan) was used. A linear gradient was prepared with 0.1% formic acid in water (solvent A) and acetonitrile (solvent B): from 20% solvent B to 100% solvent B in 25 min at 30°C. The flavonoids were monitored by a UV detector at 255 nm.

### Synthesis of 4′-*O*-methylfisetin

The methods for the synthesis of methylflavonids were described in [41]. To a solution of fisetin (122.4 mg, 4.3×10^−4^ mol) in *N,N*-dimethylformamide (10 ml) was added CH_3_I (26.4 µL, 4.3×10^−4^ mol), and K_2_CO_3_ (71.7 mg, 5.1×10^−4^ mol). After being stirred for 14 h at room temperature, the reaction mixture was concentrated in vacuo, dissolved in ethyl acetate, washed with sat NaCl, dried over Na_2_SO_4_ and evaporated. The resulting residue was separated with preparative SiO_2_ thin layer chromatography (eluent: chloroform/methanol (9/1)) followed by HPLC using a gel filtration colum, JAIGEL GS-320 (Japan Analytical Industry Co., Ltd, Tokyo, Japan) with an eluent methanol, and finally 4′-*O*-methylfisetin was recovered as yellow crystals (5.9 mg, 5.3% yield). The identity and structure of 4′-*O*-methylfisetin was confirmed with electrospray ionization mass spectroscopy (ESI-MS) and ^1^H and ^13^C nuclear magnetic resonance (NMR) [20], respectively. 4′-*O*-methylfisetin (C_16_H_13_O_6_): ^1^H NMR (399.65 MHz, CD_3_OD) δ: 3.94 (1 H, d, *J* = 3.6 Hz), 6.91 (2 H, m), 7.06 (1 H, d, *J* = 8.4 Hz), 7.77 (1 H, m), 7.98 (1 H, d, *J* = 9.6 Hz). ^13^C NMR (399.65 MHz, CD_3_OD): 56.35 (OCH_3_) 102.98, 112.24, 115.45, 115.65, 116.08, 121.45, 127.55, 147.42, 150.61, 158.57. The spectral data of 4′-*O*-methylfisetin and its derivatives is shown in Figure S1.

ESI-MS spectra were measured on AB SCIEX API-3000 mass spectrometer. NMR siganl was recorded on a JEOL JNM-JSX400 spectrometer using CD_3_OD as a solvent and tetramethylsilane (TMS) as the internal standard.

### Quantitative real-time PCR

Total RNA was isolated from B16F10 cells by using the EZ1 RNA Universal Tissue Kit (Qiagen, Venlo Park, the Netherlands), according to the manufacturer's protocol. cDNA was synthesized using the Transcriptor cDNA First Strand Synthesis Kit (Roche Diagnostics Corp., Indianapolis, IN, USA). PCR amplification was performed using Platinum Quantitative PCR SuperMix (Invitrogen). The resulting cDNA was amplified using the specific primers: GAPDH-F, 5′-ACTCACGGCAAATTCAACGG and GAPDH-R, 5′-GACTCCACGACATACTGAGC; Tyrosinase-F, 5′-TGGGGATGAGAACTTCACTG and Tyrosinase-R, 5′-ACGTAATAGTGGTCCCTCAGGT; A-Mitf-F, 5′-GGAAATGCTAGAATACAGTCACTA and Pan-Mitf-R, 5′-GTCGCCAGGCTGGTTTGGACA; and M-Mitf-F 5′-GGAAATGCTAGAATACAGTCACTA and Pan-Mitf-R. The reactions were performed for 42 cycles at 95°C for 20 s, 58°C for 20 s, 72°C for 20 s, and 31 cycles at 75°C for 10 s.

### Western blot analysis

B16F10 melanoma cells were washed with PBS and lysed with lysis buffer (150 mM Tris (pH 6.8), 60% sodium dodecyl sulfate (SDS), 30% glycerol, and 10% mercaptoethanol). Cell lysates were boiled for 15 min at 95°C and subjected to 10% SDS-polyacrylamide gel electrophoresis and transferred onto polyvinylidene fluoride membranes (Millipore, Bedford, MA, USA). The membranes were blocked with Blocking-One (Nacalai tesque, Kyoto, Japan) and then incubated with the following primary antibodies: anti-Tyrosinase goat polyclonal antibody (Santa Cruz Biotechnology, Santa Cruz, CA, USA), anti-SIK2 rabbit polyclonal antibody [15], and anti-CREB and anti-phospho CREB rabbit polyclonal antibodies (Cell Signaling Technology, MA, USA) at 4°C overnight. After washing, the membranes were incubated with peroxidase-conjugated secondary antibody at room temperature for 4 h. Detection was performed using the KONICA MINOLTA immunostaining HRP-1000 Kit (KONICA MINOLTA, Tokyo, Japan).

### Immunocytochemistry

To perform immunocytochemistry, B16F10 cells were seeded on glass cover-slips. The medium was changed with fresh medium supplemented with 10 µM flavonoid for 72 h. The cells were fixed with 4% formaldehyde and stained with the anti-TORC1/3 rabbit polyclonal antibody. To detect the TORC1-antibody complex, anti-rabbit IgG conjugated with Alexa Fluor-594 (Eugene, OR, USA) was used. Nuclei were stained with 4′, 6-diamino-2-phenylindole (DAPI).

### Expression vector, adenoviruses, transfection, and luciferase/cAMP assay

The reporter plasmids and adenoviruses were previously described [42], [43]. Briefly, B16F10 cells in a 24-well plate were co-transfected with the pTAL-CRE vector (200 ng/well) with the internal reporter pRL-TK (30 ng) in the presence or absence of the SIK2 expression vector (pTarget-SIK2 50 ng) using Lipofectamine2000 (Invitrogen, Carlsbad, CA, USA). After 24 h, the cells were treated with forskolin (20 µM) and cultured for an additional 6 h. Reporter activity was monitored using the Dual Luciferase Reporter Assay Kit (Promega, Madison, WI, USA).

The dominant negative TORC1 (DN-TORC1) adenovirus was previously described [19]. B16F10 cells plated in 6-well dishes were infected with adenoviruses (DN-TORC1 or lacZ at a multiplicity of infection of 10). After a 3 h incubation, the medium was changed with new medium that did not contain adenoviruses, and the cells were cultured for 72 h with a medium change after 48 h.

Fluctuation of the intracellular cAMP level was monitored by the PKA regulatory subunit-liked luciferase reporter system, the GloSensor™ cAMP Assay kit (Promega). Briefly, B16F10 cells were seeded in 96-well plate at a density of 5×10^3^ cells/well and incubated for 24 h and transfected with the pGloSensor™-22F cAMP-reporter plasmid (1 ng/well) using LipofectAMIN2000. After 18 h, cells were incubated with GloSensor™ cAMP reagent for 2 h, and, then, forskolin (20 µM) or flavonoids (10 µM) was added into the culture medium.

### Statistical analysis

Student's *t*-test was used to assess all experimental data in Microsoft Excel. The mean and standard deviation (S.D.) are shown.

## Supporting Information

Figure S1 **NMR analysis of 4′-** ***O-*** **methylfisetin and its derivatives (authentic).** The structure of 4′-*O*-methylfisetin was confirmed by comparison of its ^13^C-NMR chemical shifts in B-ring positions with those of other similar flavonoids owing 4′-OH or 4′-OMe with 3′-OMe or 3′-OH groups. ^13^C-NMR chemical shifts of 4′-*O*-methylfisetin for the B-ring positions, from C-1′ to 6′, are similar to those of (4′-OMe, 3′-OH)-type tamarixetin [20] and different from those of (4′-OH, 3′-OMe)-type isorhamnetin and geraldor.
(TIF)

Figure S2 **4′-** ***O*** **-methylfisetin (4′MF) does not affect the MEK or GSK-3 beta pathways.** B16F10 cells cultured in FCS-free medium overnight were treated with fisetin, 4′MF, or geraldol (10 mM) for 30 min. MEK/pMEK and GSK-3 beta/pGSK-3 beta were examined. The photographs indicate a representative set from the duplicate experiments.
(TIF)
