# Supplementary figures and images for: A Potent Inhibitor of SIK2, 3, 3′, 7-Trihydroxy-4′-Methoxyflavon (4′-O-Methylfisetin), Promotes Melanogenesis in B16F10 Melanoma Cells
Source: PLoS One. 2011 Oct 13;6(10):e26148. doi: 10.1371/journal.pone.0026148 (PMC3192784; doi:10.1371/journal.pone.0026148)

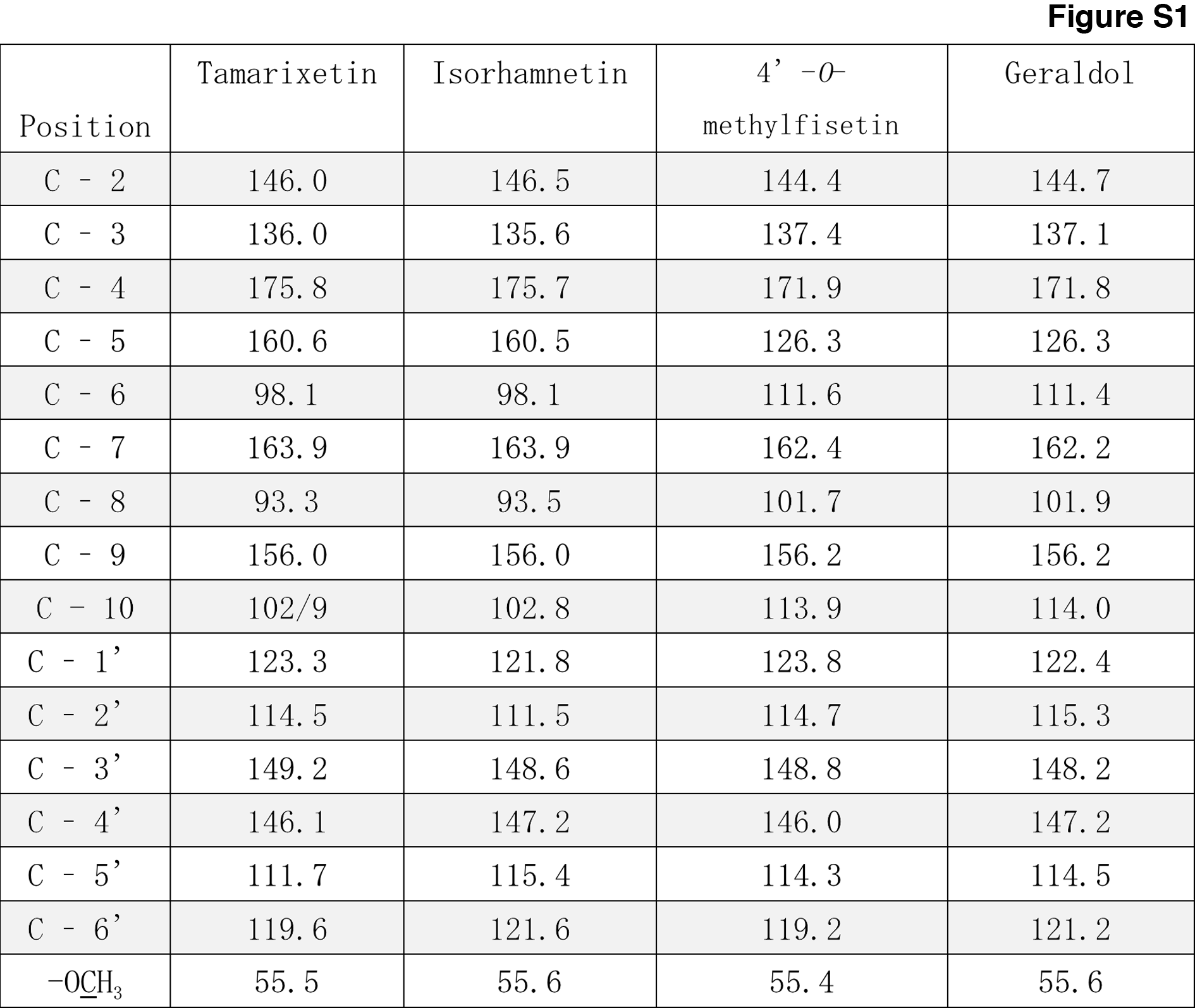

Supplement: Figure S1 — NMR analysis of 4′- O- methylfisetin and its derivatives (authentic). The structure of 4′-O-methylfisetin was confirmed by comparison of its 13C-NMR chemical shifts in B-ring positions with those of other similar flavonoids owing 4′-OH or 4′-OMe with 3′-OMe or 3′-OH groups. 13C-NMR chemical shifts of 4′-O-methylfisetin for the B-ring positions, from C-1′ to 6′, are similar to those of (4′-OMe, 3′-OH)-type tamarixetin [20] and different from those of (4′-OH, 3′-OMe)-type isorhamnetin and geraldor. (TIF) [file pone.0026148.s001.tif]

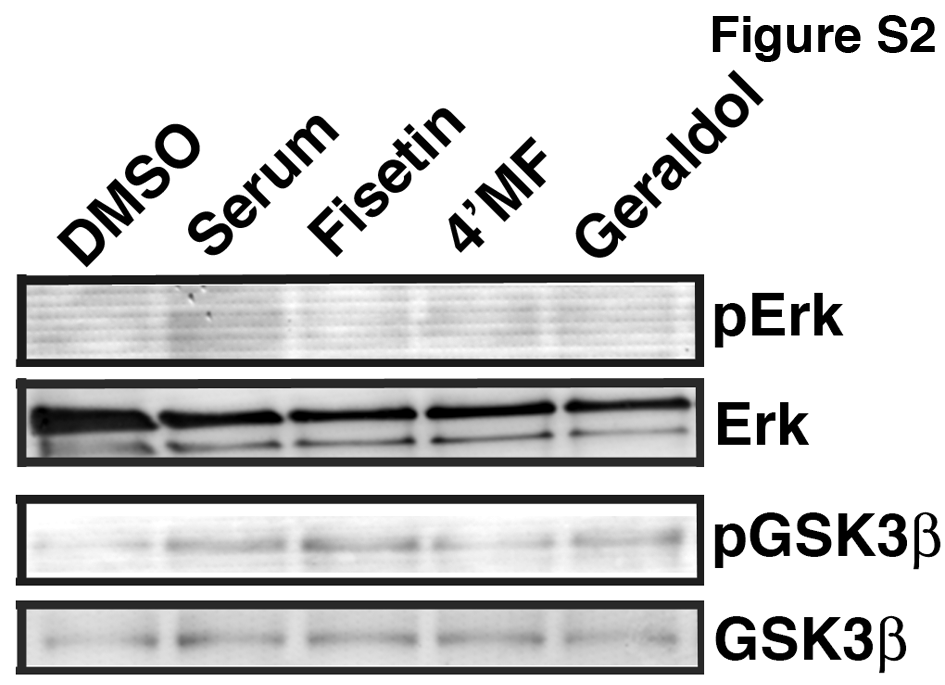

Supplement: Figure S2 — 4′- O -methylfisetin (4′MF) does not affect the MEK or GSK-3 beta pathways. B16F10 cells cultured in FCS-free medium overnight were treated with fisetin, 4′MF, or geraldol (10 mM) for 30 min. MEK/pMEK and GSK-3 beta/pGSK-3 beta were examined. The photographs indicate a representative set from the duplicate experiments. (TIF) [file pone.0026148.s002.tif]
